# Transgender Children: From Controversy to Dialogue

**DOI:** 10.1080/00797308.2021.1975462

**Published:** 2021-10-18

**Authors:** Oren Gozlan, Jordan Osserman, Laurel Silber, Hannah Wallerstein, Eve Watson, Tobias Wiggins

**Keywords:** Transgender, gender, development, puberty blockers, trans kids

## Abstract

In March 2021, Hannah Wallerstein and Jordan Osserman facilitated a live dialogue over Zoom on the subject of transgender young people, with four psychoanalytic clinicians and thinkers. The conversation draws on short essays submitted in this section of The Psychoanalytic Study of the Child as a springboard for discussion. It has been transcribed and edited for length and clarity, and is reproduced here. Questions explored include the differences surrounding gender identity in childhood versus adulthood, the use of medical interventions for children experiencing gender dysphoria, the tension between psychoanalytic neutrality and affirmation, and the ethical stakes of working in this field.

Jordan Osserman [JO]: Let’s start with a general question: what is your definition of gender? And is there something particular about gender transition versus other transformations?

Oren Gozlan [OG]: I think of gender as having to do with an internal difference; it’s not a difference between, it’s a difference within, and it’s a structural difference because there is a split internally. The conscious/unconscious, passivity/activity: we are split subjects and that creates a gap, or lack, that propels us to find a container. The anxieties about this gap are projected outside and become this binary structure of gender. This gap or split is, within Lacanian terminology, referred to as sexual difference: that which propels us to find a gendered means of embodiment. It has been difficult to speak about sexual difference in psycho-analysis outside of genitality, but it refers to an unconscious position, not a preexisting biological reality of the body. Gender becomes either a war zone of this internal difference, or a container for it. Whether hetero, homo, trans – they are all solutions for this difference. It’s sexual because we’re human, so everything involves desire, push. It always involves a question of desire.

I also think of gender as a “lint collector.” We cannot and we do not have to answer where we originated from and why, in terms of gender. That’s an impossible question. Like a lint collector, gender accrues and creates meanings as it goes along, as we go along. Things attach to it.

Laurel Silber [LS]: From my experience in clinical work with kids, I see gender taking place in the overlap of internal and external worlds. For a child to sort out a sense of self and find where to belong, there is an ongoing dialogue, asking: how am I the same and how am I different? This is an ever evolving dialogue within the child and between them and their attachment figures, (including ancestors) and their social context. I agree with Oren’s mention of a “lint collector” – I liken it to Adrienne Harris’ concept of gender as a “soft assembly.” Gender is ever reconfiguring, recontextualizing from various experiences, ideas, and feelings. Where I would shift in emphasis from what you said, Oren, is that I find in my work with children that gender lives in an exterior space, as well as the interior.

OG: I agree with you, we cannot think about gender outside of the social. It has to do with how we’re read, with recognition. Like Winnicott saying there’s no such thing as …

LS: As a baby. Exactly.

OG: My point is that the split is internal: the difference is internal, and gender is a secondary construction, so the split becomes gendered.

LS: I came upon a conclusion that I think might be aligned with your view; I titled my paper on this subject: “Locating rupture encrypted in gender.” Similar in the sense of thinking about splits, rupture, and differences that then become gendered.

Tobias Wiggins [TW]: I really appreciated reading everybody’s definitions of gender. There is something about gender that seeks constant attempts at definition that will always fall short. I agree that there’s a combination of the social and the relational; it’s about loss, memory, identification, melancholia, fantasy. I like Susan Stryker’s definition; although it’s not psychoanalytic, she says that “trans” can be defined as moving away from an unchosen starting place. I like that definition because it names a specific trans experience, but also speaks to a broader experience of gender that we all have. Origins are never chosen and we always move away from them, and I think gender has a big part to play in that.

In my own writing, I don’t think I’ve ever really defined gender. In part that’s because there has been such a fixation on trying to define gender in relation to trans experience throughout history, which is linked to a history of pathologization. Because of that, I’ve become interested in people’s reactions to gender variance, or what they understand as gender nonconformance, more than in defining the term gender itself.

I’ve turned, instead, to the issue of perversion. In the Lacanian sense, everyone with a neurotic structure has a relationship to perversion, a desire for what has been lost in castration. In cis people’s fantasy, trans may bring up an idea of what has been lost, and that can generate an anxious reaction that, in turn, creates this need to constantly define or understand gender with certainty. Many of you have named this need for certainty around gender – I see that really come into play in trying to define trans.

Eve Watson [EW]: I find myself thinking about the connection between gender identity and sexual difference, which is so often conflated. Psychoanalysis tries to retain those as different: sexual identity being an unconscious process that happens in the early years, and “genderization” as social and cultural processes, and having an iterative aspect to it. These do go hand in hand and operate together, to an extent. But, why do we go through a process of sexual differentiation at all? Maybe we should talk about that question. Psychoanalytically, things all get to be a bit “too much” for a child at a certain point. Inchoate drives meet with processes of socialization, and the solution is an identity which has a sexual aspect to it. Why does our identity which comes into being in the first six years or so have this sexual aspect to it? Of course it has to do with social and cultural processes, and it also has to do with the particularities of human anatomy, as they are taken up socially and culturally.

We come to be something because we’re not something else. That’s how the process of identification occurs, which is destined to be incomplete and unsatisfactory. And one of the ways that we do that is through the singularity of our own familial circumstances, through questions of anatomy, and through the particular way in which we are impacted by language and culture.

We could also ask: why have there only been, more or less, two genders up until now? Why has it taken this long? Those are the kinds of questions I find myself asking.

TW: Regarding your last question Eve, I would underscore that there are long histories of people whose gender falls outside of a binary. For example, the belief that there are only two genders is tied to European expansion and imperialism; the gender binary is a colonial construct. The lives of gender non-conforming people have been hidden, and sometimes violently suppressed, but we have always existed. This is also relevant in relation to the trans child. In *Histories of the Transgender Child* – one of my favorite books on this topic – Jules Peterson asks why we imagine trans children to be a new phenomenon. You see this reiterated in the media all the time, that trans kids are “new.” It’s saturated with anxiety – *why are there so many trans children*?! Peterson points out that in the context of Western medicine specifically, our conceptualization of “transgender” as a term actually rests upon the lives of gender non-conforming children. Psychoanalytic theories of gender, like Robert Stoller’s for example, drew upon the bodies of trans children (broadly considered), including intersex children, whose morphology fell outside of binary expectations. So, what I want to point out is that while trans children are not novel, perhaps what’s noteworthy is that they’re considered to be novel.

JO: Some of you noted that childhood presents particularly urgent questions around identity and self-hood. Eve challenged the notion of sexual development itself, pointing out that Freud posed sexuality in terms of query and the “sexual researches of childhood.” Others emphasized children’s capacity to define themselves and their gender, calling into question assumptions we make about children’s immaturity.

What do you think the status of childhood is, in relation to adulthood? What, if anything, is the clinical distinctiveness of childhood in comparison to adulthood? Relatedly, should we think of adult and child trans identifications differently?

LS: I would say there’s major differences between childhood and adulthood. The child works through dependency toward relative independence or interdependence, back and forth, within an attachment context. That, I think, distinguishes the needs and responsibilities of the other in the circumstance of childhood.

I was interested in Tobias’s comments (from his essay) regarding Elisabeth Young-Bruehl’s notion of childism. I’ve thought about childism in terms of our society’s elimination of interest in the inner world of the child, both in the child mental health world, and in the culture at large. I think childhood is really in crisis right now in the US, from a mental health and social perspective. Kids, teenagers, are marching for their lives, terrified there’s no gun control, that the environment is deteriorating – all these issues raise the question of the future. You’ll hear it in Greta Thunberg speaking: kids are terrified for the viability and sustainability of their lives and their childhood. Myself and my colleagues are getting calls nonstop for mental health care for children, and Covid brings an additional layer of trauma. So in my view contemporary childhood is not in good shape. This suggests further stress and challenges to organizing a sense of self.

As for your question about the transgender child and the transgender adult, I just wanted to say that, with regards to mental health, I do see these as very different circumstances, requiring different approaches and ways of thinking. A child therapist enters a formative system of relationships in working with a child. Therefore, multiple parts of the field, if you will, become part of an intervention (which I take up in a bit more detail in my individual essay). And while adults are also embedded in a social context, developmentally speaking, more of their identity is internalized.

OG: There’s no question that children are different from adults. They have a short view, because they live in the present, they don’t think about the future and they do not have a past. The child also depends on our care, and that places us *in loco parentis*. In the case of the analyst, that involves a desire for the child to be safe, and in the context of gender, it may mean that the analyst wants to delay the child’s transitioning to avoid the possibility of regret. But we must be very careful when we talk about “the child.” It’s in the realm of the hypothetical and universal. Children are not the same, so we have to ask: what child are we thinking about? Children, and trans children, differ. Some will regret their transitioning, some will find relief in their transitioning, some will be excited by their transitioning. One of the questions that the analyst should ask themselves is: can the child tolerate regret, and can the analyst tolerate the child’s regret?

Whatever the risk, whatever worry the analyst has, I personally do not think that we should think of the clinic as a prophylactic.

Hannah Wallerstein [HW]: Can you say more about what you mean by that Oren?

OG: We cannot predict or prevent the future. We’re there to help the patient – child or adult – understand that we’re curious, and in turn to help the patient also become curious about themself. Regarding our anxieties about the future, that’s something that relates to our own anxieties as analysts.

TW: I like your statement about the clinic not being prophylactic. I think “the” child is a nice way to put it, because it is a figure, it is a container for fantasy; we’re not actually talking about real children when we’re talking about the figure of “the transgender child.” One thing that occurs on both sides of the debate – if we can create a binary like that – is the question of the child’s happiness with the transition “results.” If we give hormone blockers to a child who is gender non-conforming, can we predict that they will turn out to be happy, to have no regret? You see this clearly deployed in the anxiety around the UK High Court case (*Bell v Tavistock*) where someone who identified as trans no longer identifies as trans, and is quite unhappy with what occurred, and that unhappiness can justify restrictions for others. But you also see the promise of particular outcomes used amongst those who advocate *for* access to hormone blockers, and deploy statistics about results: x number of trans children ended up with no regrets, or are quite happy with their transition, or suicide rates have decreased this much. You see this deployment of statistics back and forth between both sides. It’s ping pong with positivism. I would say this is a kind of obsessional response to a question of difference and uncertainty – there’s a deployment of a rule, and the idea that we can know for sure that trans children will or won’t be happy.

I wonder what would be possible if we allowed space for trans people to have a much less rigid experience of transitioning: with the understanding that you move through transition as one moves through any moment of change, and at the end you can’t split between everything being good or bad, or having a positive or negative outcome.

HW: It seems we are speaking about the aspect of childhood that concerns dependency, and anxieties regarding the protection of kids. Another part of childhood, that distinguishes it from adulthood in my mind, is a particular relationship to development. Some of you pushed back on developmental ideas or emphasized continuity. But I’m curious how you can conceptualize childhood without an idea of development?

OG: It’s not that I conceptualize childhood without development, but rather that development is uneven, constituted through interaction, disruption. There isn’t an ideal “natural” path which we interrupt with hormones, or a different representation of gender. Human development is constituted through interference all the time. What I am challenging is the naturality of nature, an ideology that can be quite profound in psychoanalytic thinking.

EW: Agreed. The impact of language means that the idea of development as some kind of linear, hierarchical progression is denatured, if you will. But that’s not to say there aren’t aspects of development that occur! Clearly children develop physically, psychologically, emotionally, cognitively and so on as they progress through childhood and into adolescence.

One of the things I’m provoked to think about in this discussion is the clinician’s counter transference. Of course there are responsibilities and duties in working with a child that the clinician adheres to. But the possibility of regret around a choice, or a decision made by a young person … I suppose I’m a little bit troubled by this because there’s always regret, there’s always a risk, there’s always the absence of guarantee around any kind of decision or choice, and that’s exactly what we permit in allowing somebody to come along and speak, and speak freely. To speak to the ambivalence that exists, around a path that they may be moving toward and ultimately taking. Indeed we often work with people to untangle them from the oppression of regret.

Regret is one of the most serious affects, but it’s also one of the most useless affects. There are affects that are very important, that we work with very carefully, but regret is the super ego’s fuel. It is how the superego is expressed. One of the things we do is try to allow some space between the tyranny of the superego (and its instantiation in regret) and decisions made for whatever reasons they’re made. So I see this as a very important discussion. We hear things all the time clinically – we may even think, oh no, that’s not going to go well! But it’s not for us to get in the way of this, it’s something that we help the patient to work out for themselves.

HW: I want to push back a bit on the consensus we seem to be coming to regarding regret. What has been said about the futility and dangers of attempting to protect our patients from regret makes perfect sense to me when it concerns adults and older adolescents. But I’m not so sure when it comes to children. Perhaps a better word than regret, is responsibility. I think of childhood as a time when one is not fully responsible for oneself. And for good reason! If babies could, they would likely murder all of us in moments of distress. It seems to me to be crucial to development, and, ironically, to the capacity to assume responsibility for oneself later on, to have a period where one is protected to some degree from such responsibility. This question of responsibility comes up in conversations about puberty blockers or early uses of hormone therapy. An argument can be made that children and young adolescents should be protected from making decisions that alter their bodies for a time, until they are more independent. I would love to hear your thoughts on this.

OG: For me, the question is the nature of the child’s demand. Children have different solutions and different demands. When a child comes with a demand around gender, they’re not necessarily immediately demanding medical interventions, or surgery. Children have not developed secondary characteristics and so the question of hormones or surgery is not the question here. Often we’re talking first about hormone suppressors. From the literature that I’ve read and particularly the research done in the Netherlands, I think the anxiety around hormone suppressors is really not proportionate to the reality of the intervention. I would add that for the analyst there is an abiding uncertainty about what peopl e – children or adults – really want, versus what they say they want. That uncertainty is what psychoanalysis teaches us. But also, there is the question of suffering, and this question pertains to the capacity for livability and being in the world. My patient wants to be in the world in a particular way. Are we sure what the person wants? That question belongs to the patient, as the analyst cannot reside in certitude or knowledge. What is clear, is that something like a letter, usage of desired pronouns etc., is a small thing in the process of transitioning.

JO: Perhaps it makes sense here to also ask your thoughts on the significance of puberty, given puberty blockers were brought up. Jean Laplanche makes the point that first we have infantile sexuality, then all of these hormones rush in during puberty and have to reckon with that preexisting sexuality. So there’s a kind of cross-contamination of the biological effects of puberty with our phantasmatic infantile sexuality. I’m interested in how you might think about this tension between conceptualizing puberty biologically versus psychoanalytically, which comes up in discussions about the possibility of medically delaying puberty.

TW: One interesting thing that occurs in the psychoanalytic literature is that trans people, along with other subjects who were rendered clinically perverse, were considered to be arrested in development. So there’s a kind of belatedness to trans subjects in psychoanalytic literature and these threads, although not as overt today, still continue in the ways that trans people are talked about. On the one hand trans people are constructed as belated, stuck and arrested, but on the other when there’s a request for stopping, for waiting, for being arrested or stuck in the use of blockers, there’s a strong resistance. Eve brought in the topic of counter-transference, which we all talk about in our articles to some extent, and I think that this might be a useful place to stop and pose a question about its influence.

OG: No doubt, adolescence involves rebelliousness, and part of the rebellion that it takes is through gender. But Jordan, I think you were raising some questions regarding the turmoil involved in adolescence, and what it means for transition?

JO: Yes – some of the arguments against puberty blockers propose that puberty is a developmentally necessary sexual and identity “shake-up”; that this moment must be experienced before an identity can be decided upon.

OG: That’s an idealization of a linear progression of development – the idea that something predetermined has to happen during adolescence, that you cannot confront an alterity or an interruption. To me what’s more interesting is adolescence as a state of mind. The kind of anxieties that we see in the field around transitioning, and the regret and the worry about the future, those I think are articulations of the analyst’s adolescent state of mind. In other words, adolescence can be seen both as a developmental stage and as a s*tate of mind* anyone can enter into, in which there is difficulty distinguishing between wrecking objects in one’s mind and building the world outside. The analyst’s state of mind is not immune. You’re there sitting with someone who is making a demand, pushing against you, your ideas, perhaps your gender – you may be very distraught and you may have a reaction, a counter- transference reaction. The only thing the analyst can do is to tolerate the anxiety, watch it unfold and see, with the patient, what the demands mean. I think of adolescence along the lines of Kristeva, the adolescent state of mind. And that is not just a property of the adolescent.

EW: On the subject of adolescence, I wanted also to add to Oren’s ideas the importance of separation, and the striving toward independence. I like Adam Philip’s description of adolescence as the adolescent’s wish to murder the other! It invokes the kinds of passions that are at stake here. I tend to think of the three great passions: love, hate and ignorance. In thinking of the importance of separation and the desire for independence that is characteristic of adolescence, and which underpins the development of a sense of self including a gendered self, we can think of the fundamental demands the adolescent is working at. These demands can include a demand for recognition, for acceptance, for love, and even for hate and punishment. There are other contexts to be taken into account. We are never just isolated in the world, we’re always in and deeply impacted by our relationships with others. So the question of responsibility – I’ve never stood in the way of a young person seeking any intervention they want to get. There is a problem with the availability of interventions in Ireland, for those seeking to transition – we have a dearth of services, and that’s a whole other problem. But I suppose one of the challenges I’ve encountered with young people is a resistance to speaking about psychology, about the psyche, about personal history and its significance. It’s connected to the kinds of information and knowledge that young people are accessing, which emphasizes physicality and the body as a site of change and demotes the psychological factors at stake. I see this demotion as misguided, as any change involves a mental and emotional side. In fact, it can be the most significant part. I wonder if others have encountered that? I’m very curious, but sometimes I’m the only person who’s curious in the room. Sometimes I’ve been successful with fostering that curiosity with my young analysands, and frankly sometimes I haven’t.

TW: Oren, I want to ask a follow-up to what you’ve just said. You, and Eve, both use the word “demand.” You say that the child or adolescent is making a demand and I believe you’re using it in a Lacanian sense. But as a Trans Studies scholar, there’s this little red light that goes on in my head about saying that a child’s assertion of gendered being is a “demand.” The demand might be “I’m a boy” or “I’m a girl,” or asking for blockers, or “I’d like to wear a dress,” right? I’m wondering if you could elaborate on your choice of the word “demand.” I’m concerned that it could be seen as framing the gendered assertion in a negative light, as if there’s an entitlement or perhaps an unsubstantiated or thoughtless request for something that is experienced as demanding.

EW: I remember this from day one in my training: “behind a conscious demand that is made, there is an unconscious wish or an unconscious desire. Beware taking demand at face value.” So in a very general sense Toby, that’s what we’re referring to here, the importance of not stopping at the demand. That it doesn’t go far enough, that we must investigate the unconscious desire somewhere behind the overt or conscious demand that is made. It does require some explanation and contextualization. I can understand how it would be heard in a negative way. In a colloquial or ordinary sense, we don’t think of demanding people in a positive way, but in psychoanalysis demand is part and parcel of the work. An analysand arrives, for example, with a demand to find an answer to a problem or a symptom and that’s important. This is a beginning and is a pathway to other unconscious factors to be discerned. OG: For me, transitioning is also a demand placed on the other to transition, it’s not just an individual transitioning. I demand of you to refer to me in a certain way, to relate to me in a certain way. There’s a series of demands that are also involved in human rights. I feel it’s my right to be called a certain way, and that’s a demand upon the other.

LS: I was thinking, for the purpose of this conversation, I might conjure up a scenario with a real child.^1^ Because, as Tobias said, we can make myths about children so readily, and project onto them. Looking at a particular child’s “demand” may open up to the possibility of, as Eve mentioned, investigating, or considering; “What this might be about?”

A father of two young children, a toddler and an infant, dies in a sudden tragedy. The toddler began working through the trauma in child analysis through behavior, play and language. As the infant developed and began to speak, she started asking for the father, wanting to know where he was, looking for the father, asking when she could see him and wondering if she might die and then see him. As the infant became a toddler and began to react to pictures and stories about him, others shared memories of him and the longing intensified. The struggle with his absence was shared with the family, but there was a feeling of difference the now-toddler was grappling with – others got to know him, and she did not. During this period she stated to the family, “I a boy.” In speaking to the mother about this, I both counseled her to appreciate the child’s “boyness” (she can be anything she wants to be) and to wonder about all that this comment may be communicating, including the child’s efforts at managing grief. It is hard to find words to communicate about longing and absence. One could conjecture that, in this child’s search for her father, she is him, she is like him, in order to find him in her (for herself and others for whom she wished reparation). To feel her connection to him in her body and grieve him, as others in the family were in various stages of doing. In addition, it occurs to me that the world was nonconforming to her needs – imposing a tragic loss. In asserting “I a boy,” the child may be also communicating something of this other kind of nonconformance. So, it seems to me that, referring back to Hannah’s comment about the protected space of childhood, one of the things children require is the help from parents to unpack things, including gendered statements. Much of the developing capacity to name, sort out, and bear difficult feelings depends on the adult’s response in the bi-directional, on-going communication.

JO: It sounds like you’re saying that the quality of curiosity that Eve referred to, is something the parents must offer as well.

LS: Absolutely. One could refer to that as a responsibility, in the context of the earlier part of the conversation.

TW: It’s also so important when listening analytically to think through counter- transference, and in particular the difficulties that come up in the face of gender difference. Your story is quite captivating and layered regarding loss and identification. However, I can also see how it could be used in a kind of transphobic way: the child experiences a trauma and because of the trauma, they have gender nonconformance. That type of story’s been reiterated in a lot of pathologizing psychoanalytic writing. We need to listen carefully to the child in that situation, and that’s why I turn to Young-Bruehl’s childism. The question that parents should ask, and clinicians should ask, is: am I using the child as an extension of my own subjectivity? Is there a way that my own anxieties are showing up in how I am conceptualizing the articulations of the child? If the child assigned female at birth says, “I’m a boy,” … I think that it’s very easy to slip into one’s own fears about gender, and then create what Young-Bruehl calls a kind of prejudicial response.

EW: I have a question for Toby. I’m so glad we’re having this discussion, because I would like to ask you to help me understand how the linking of trauma to some kind of outcome – in this case, the assertion of an identity – is transphobic. I suppose I’m coming at this from the Lacanian framework, where the very encounter for every single subject with language is, by definition, traumatic. It’s how we deal with that, which is determined structurally in terms of whether we end up neurotically inclined or psychotically inclined or perversely inclined, and then variations thereafter as well. So trauma is always there. I would love it if you could help me to understand precisely what you’re getting at there, because I think it’s really important, when you say that the connection between trauma and sexual identity or gender identity would be transphobic.

TW: Well in the case example, it’s a secondary trauma – meaning it’s an unexpected, disruptive event that the subject cannot mentalize; in your example, I believe you’re talking about a primary trauma, the quotidian trauma of becoming a subject in language. But the way that it is transphobic, is the way that it’s used subsequently, to turn the subject in question away from gender nonconformance. The implication is that if we address the underlying trauma, then the person will no longer be gender non-conforming. That’s the turn in the psychoanalytic literature: the gender non-conformity becomes the pathology, the thing that will change when the trauma is resolved. But I do think that there’s a way to talk through trauma, and certainly trauma does have to do with the development of gender and with sexuation. Again, it’s how that theory is deployed within psychoanalytic literature, within the clinic, that ultimately seeks to remove transness, where transness and gender nonconformance is seen to be the worst possible outcome.

EW: So it’s a hetero-normativising, if you will? And the deployment of a normative, binarizing approach that ends up being transphobic. Certainly I’ve read many accounts of that.

TW: And in turn, it’s necessary to think about how we could write about both gender and trauma without pathologizing trans experience. Oren does this when writing about the relation of gender to trauma without assuming gender nonconformance is the worst possible outcome. But also making clear that for everyone, gender is non-conforming. The idea of a conformance to any kind of fixed outcome is a fantasy, a cisgender fantasy that I think causes a lot of trouble for trans people.

HW: It seems that what you’re pointing to Tobias is the use of interpretation coercively, where links or hypotheses are made in order to obtain a certain normative outcome.

TW: Yeah, but I don’t think that it is ever very intentionally coercive either.

HW: Sure, yes.

TW: It’s often unconscious and again, there’s the issue of counter-transference. Gender appears differently for cisgender people because there’s a kind of taken-for-granted-ness about that naturalization of gender. And so when someone appears in the clinic who is different from what we’re accustomed to, it’s quite disruptive, and for the clinician who hasn’t taken gender into consideration, it highlights aspects of their own gender that they haven’t had the opportunity to unpack, and then that work starts happening in the transference and counter-transference in a way that can be harmful for trans analysands.

OG: I think we see a lot of the same kind of alarmist views in recent theories that propose a kind of “Russian doll” idea of gender, where if we look under the layers of gender non-conformity, we will find a kernel of suffering that explains it. This insistence comes under the guise of taking a “deeper” approach to gender by focusing on the “why” of gender. The assumption being made is that if that underlying symptom is removed, we can change the course of gender. But I think when we pose the question “why transgender?” analytically, we cannot ask for an origin. We can only ask what the patient wants to do, how they wish to present themselves and how they came to this. The question of “why” transgender is answered over time and in different ways. We have a series of narratives we use to try to stabilize our position, including: “I was born this way.” We do have to ask “why,” but not in a sense of a “cause that causes every cause.” It is rather a phenomenological question, analytically speaking. If we ask it in this way, it is a question that applies to every life changing choice we make. It changes everything about our lives in every category. The emphasis should be placed on the *narrative* that is constructed in response to the question: “why this solution?.” It cannot be a simple “because.” I tend to think of these underlying conflicts as very unconscious and inaccessible. All we can see is their dispersal in the present. Like with a dream, one can never reach the “navel” of gender. Because of interiority all one can get to is the underlying *phantasy*, not the conflict: behind each conflict lies another conflict in a slide toward infinite regress. This is a blow to the human, who will always be subject to this gap and can never be whole.

LS: I think Oren your comment about so much being inaccessible is right, and I think it is even more true when someone is two and three – the way experience is accessible is through play and behavior, not so much through language. And so that’s again where it is very important to help parents think about what the child may be struggling with, that is being expressed in actions, and to help parents translate that into language. I feel in a contested place around this, because to open up the question of what the child’s gendered assertion may be communicating for this mom in the example moves it away from gender per se, and then that can be seen as transphobic, or pathologizing gender non-conformity. In my view, this three-year-old uses what she can in order to communicate what is inaccessible to her – what she’s struggling with, what she’s feeling – in an attempt to have it reach her mom to be recognized. Gender, here, may be in part a vehicle for communicating all sorts of things that can be opened up. I think it is so important to interact with a three-year-old’s communications in a way that enlarges and expands on them. When I do that, I don’t think I am acting in a transphobic way; I am curious about a young person’s attempt to communicate something that is difficult to articulate. I appreciate that there’s ideas I may project onto the child, and that you have to be mindful and think about that. There have been errors made in the history of psychoanalysis that need to be recognized. But in the example I shared, I don’t think of this way of working as transphobic. I don’t know if that’s what you were saying Tobias.

TW: It’s interesting what starts to happen when I bring in the word “transphobia.” I think it causes some panic about harming patients through one’s own prejudice. I like to think about transphobia working just like any system of oppression – it has a psychic life and is going to appear in any clinical situation. Not turning away from that quotidian violence is very important. I didn’t mean to imply that speaking about the possible meanings in the child’s gendered articulation was transphobic. But there are many ways that transphobia could appear when a child assigned female at birth says, “I’m a boy.” As Young-Bruehl writes, prejudice makes others an extension of our subjectivity. This happens all the time. It can be very acute, like in the example of abuse, but we are constantly projecting ourselves onto others defensively, and gender is an arena where this plays out a good deal. So as a clinician, the first step would be to think through potential counter-transference reactions. But I agree it’s important to make space for all the meanings that might lay behind gendered speech, and not shut down possibility.

LS: I think that in addition to the panic over being called transphobic there is another worry about collapsing into binaries that eclipse the space for exploration. To explore is a clinical imperative and if that is considered “turning away from gender” (and therefore transphobic) another binary is created – to turn away from gender, or to turn toward it. Exploring a patient’s mind should be in addition to looking within to explore one’s own prejudices, as you are underlining. The exploration needs to be non-linear, and as Winnicott cautioned, not seek to resolve paradoxes, but rather reflect on them and hold the tension of them.

HW: Continuing this conversation about clinical approach, I’d like to turn to the concept of affirmation, and what is referred to as the “affirmative model.” Each of you take this up in your essays, sometimes in opposing ways. This led us to wonder: What do you mean by affirmation, and what do you mean by neutrality? For instance, Oren, you speak about “wild affirmation” – is there a non-wild affirmation? And Eve, you use the term affirmation positively in reference to the singularity of each subject. Is there a difference between this and other types of affirmation?

TW: I just want to echo that I was really taken aback by the word “affirmation” and how it appeared. I’m glad that we’re talking about the word and why there’s opposition to it, from psychoanalysis, which I’ve heard before.

EW: I agree completely with you Toby. This is a really important component of our discussion today. I’ll try to speak to what I was aiming at in my written piece. I was distinguishing an affirmative approach from a neutral approach when it comes to the position of the psychoanalyst. If the psychoanalyst works with an adolescent on the basis of having taken up some kind of affirmative position, or the opposite, which would be a negative position, that is not a position of neutral listening. So I hear in the affirmative approach an already established support for the analysand’s wish to transition, or to follow some path in relation to their gender, whatever that is. I’m fascinated to know how that could be aligned with a psychoanalytic position. Now we have already recognized importantly in our discussion today how difficult it is to maintain a neutral position in this area broadly. But I suppose it gets back to the question: do we take at face value what the analysand says at the beginning of a therapy? Or do we wait to hear whether there’s more to it? And can we do that if we take up an affirmative position from the get go? I don’t see how, and I would worry about that.

TW: One thing that I noticed in the written pieces was that affirmation seemed to be linked with foreclosure; that if we affirm an identity, we foreclose the possibility of exploration. I recently read this really lovely piece by a mother of a gender non- conforming child who spoke about trying to take a “watchful waiting” approach.^2^ I know that none of you are advocating for a watchful waiting approach, but it’s useful to bring it up, as the advice the mother was given was to try and create a space for the child to play with gender without affirming the child’s assertions. But, as we all know, not responding is a type of intervention. So the child was saying “I think I’m a girl,” and the mother would not respond, she wouldn’t say “oh ok you’re a girl, tell me more about that” or “we’ll use she/her pronouns with you, since you are asking us to,” and the child sunk into themselves, became more and more depressed, and started to psychically disappear. The child was saying “I have this thing that I want to show you,” and instead of mirroring that child’s experience, or maybe taking it in in a Bionian way, helping the child metabolize it, and giving it back to be played with, there was a wall being assembled between them. And as soon as the mother said “OK, you are a girl, I hear that you’re speaking about yourself in this way,” the child started to really blossom. Recognizing the child’s assertions of being isn’t necessarily foreclosing all the ways that can exist and appear for the child. Choosing to acknowledge someone’s gendered assertion doesn’t close down the possibility that it could change over time. So, I want to differentiate between affirmation that forecloses possibility, and affirmation that is a recognition of existence.

One other component that isn’t taken into consideration is how, if you are an individual who has gender congruence or gender normativity, your gender is affirmed constantly. Most people walk through the world with a baseline of being seen as the gender that they feel themselves to be. The request for affirmation may be just a request for a baseline that most subjects receive. Perhaps an affirmative psychoanalytic approach is one that takes into consideration the social container that trans people find themselves within, and the trauma of misrecognition. Maybe affirmation shows awareness of transphobia and an openness to having a conversation about it. I don’t think it necessarily forecloses the possibility of exploring the meanings connected to gender.

EW: I agree with you Toby, and that’s not actually what I’m getting at, I’m getting at the clinician who takes at face value and doesn’t ask any questions or enquire further about a child or adolescent’s gender assertions. I’m thinking of Ehrensaft in particular, who asserts that the child is always right and is not to be questioned, and the clinician is guided by the child’s assertions, as opposed to allowing for the possibility of that to be played with, to use the language of Laurel today – to be not an end point, but the beginning of a discussion.

TW: I’ve read Ehrensaft’s work, and I don’t see it foreclosing possibilities for discussion, but rather affirmation as a baseline for approaching that exploration.

OG: While I agree with you Toby that it doesn’t make sense for an analyst to argue with or refuse any patient’s gender identity, when I talk about the affirmative model, I’m not referring so much to what the analyst says to the patient about their gender per se, I’m talking about the mind of the analyst. What I read in Ehrensaft’s work, regardless of whether she affirms the patient, is an idealization of an “authentic gender.” I see this as very close to Lemma’s work, in that there’s an idea that if you take out all the layers, you’ll find this kernel of original gender. That to me sounds very much like the narrative some transgender individuals construct of being born in the wrong body. These are fantasies that foreclose something – they foreclose the effervescent nature of sexuality, something in excess of gender. So I think Ehrensaft’s model does foreclose something, not in the sense of what is said to the patient about their gender or pronouns, but in the way it thinks about the “kernel” of gender as something preordained. The term “authenticity” is problematic. it leans upon certitude. The future then also becomes very concrete. If you have an origin, then it has a destiny. And I think about gender differently, as inseparable from the enigma of sexuality. In this way, gender is not tied to questions of reality or truth.

LS: This conversation about foreclosure is so interesting because it gets back to a psychoanalytic state of mind, and the effort to maintain an open mind to what is going on for a particular patient. It’s so key for our job to be able to keep our minds open and also convey this to our patients, to look at the contradictory ways things feel. A child could say one thing and behave a different way, and the parent says another thing, and we have to bring together the multiple pieces. But I agree that any time a child asserts that they use this pronoun or this name, what is most important is to join them where they are and in how they are experiencing themselves in gender, as in any other dimension. That, to me, is the beauty of psychoanalysis – meeting the patient where they are, while also opening up possibilities within it.

This conversation is also making me think about the old analytic attitude regarding action vs. symbolization. Particular types of communication, like words, tend to be privileged over others, such as behavior or action. Again, it’s a question of openness, of being open to however something is communicated.

HW: Jumping off the question of action, we wanted to ask how you think about the analyst’s role in relation to medical intervention. Both conceptually but also practically – do you write letters supporting puberty blockers or surgery? And how do you think about the urgency that surrounds questions of medical intervention?

JO: Yes. And what is the line between good medical practice and medical gatekeeping?

OG: I think gatekeeping is a problematic idea, but individuals come to our offices with clear wishes and demands about their gender, and we are dealing with the question of human rights, which really limits the analysts’ neutrality. There is no neutral point from which to engage with these questions. Although the way I respond to adolescents who request letters is of course a case by case, I do from time to time write letters, but the kind of letters I write, if I write any, do not say that this individual should start hormones – I instead speak to what I see in terms of the patient’s capacity to make decisions for themselves. That’s something I can comment upon. And their decision is theirs.

EW: In Ireland, psychologists and psychiatrists write letters to services and medical clinics, so working as a psychoanalyst I could write a letter but it would be meaningless as letters are accepted only from psychologists and psychiatrists. I’ve been asked and I’ve clarified with analysands very early on that I don’t do that, in case that is an expectation of the therapy. I think it’s important to clarify with analysands that I don’t do that and that’s part of their decision in choosing to work with me. Whether they want to continue ethically, that is really important.

JO: Eve, I’m curious, if it were the case that psychoanalysts in Ireland could write such letters, would you see your position shifting, or would it remain the same?

EW: Well as a psychoanalyst I’m loath to get involved in patients’ lives outside of the clinical room. That’s not to say I haven’t in the past and it’s an interesting question. I’m thinking specifically of teenagers as I don’t work with children. You know, once we take up the position of advocacy and support and writing letters, we are in a different position from an analytic one. The work may call for that from time to time. I’m not against that, but it would need to be measured against the cost transferentially, how the transference is impacted by it. I’d have to think about that the way I think about any move that shifts me from the position that I occupy in the clinical room. But it may be appropriate, it would depend. I suppose it is something important to think about, I would love to hear about how others manage that.

TW: I’m not a clinician, but I would write letters for people if I was a clinician. I think it’s a very simple and important act of support for a community that carries an enormous legacy of being denied basic healthcare. Whenever this question is asked, I often hear responses that are very hesitant, even though there are clear standards of care for prescribing hormones or blockers that you can access on-line, and even though it’s quite easy to write a letter and to follow the standards of basic healthcare for trans and gender non-conforming people. I don’t see this interfering with a psychoanalytic approach. In fact, I would also add that there’s such a great opportunity in letter writing to explore what comes up for your analysand in the transference – perhaps the meanings behind working with a clinician who holds the power to grant healthcare services, both in terms of the tangible impacts and also in terms of the fantasies surrounding it that are particular to your analysand.

LS: The systems as they’re set up, the fact that there is gatekeeping and letters and processes put in place to create hurdles, the whole system is problematic, and working in this circumstance is also problematic. To be able to own and acknowledge all of this, seems important, regardless of whether you write a letter or not.

TW: Yes, like you need to adhere to the criteria of the DSM in order to access hormones.

LS: Right, and then kids come in having learned scripts about what to say to the doctor to get that “OK,” to get that pass. So the whole thing becomes inauthentic, just jumping through hoops, and the importance of sitting with and bearing witness and representation and real process is missed. It obstructs the act of thinking together about what feels right, and about how to live with ambivalences about decisions we make. It’s really very hard. I think a lot of child therapists say, “oh, I can’t possibly work with trans children, I have to refer them out,” which is a shame, because it further separates gender out, as a specialized and narrow field. In addition to implicit prejudices, I think the systemic issues make it hard to maintain an open mind.

TW: Maybe that’s why it’s important to just write the letter. I watch analysts hesitate and pursue extensive talking first, but then trans people are being restricted basic care. That to me is the affirmative model: provide the baseline of being able to exist, and then maybe the trust can be built for real talking to occur – for the complications, the ambivalences, the regret, the bad feelings along with the good feelings. When I was doing peer support with transfolks, I noticed reticence to talk about parts of transition that didn’t feel good, parts of a surgery that didn’t line up with expectations, not necessarily regret, but disappointment in the body, or disappointment in gender. There’s this truncating of trans people’s bad experiences, which also creates a field for the law to step in and prohibit certain possibilities for trans existence, as has occurred in the UK case. But the reason why there is reticence is because historically, if trans people didn’t have a narrative of complete satisfaction with their chosen gender, they would not be given access to care, and more generally they would be seen as further delegitimized.

HW: Our last question concerns ethics. It was fascinating that every single one of your pieces ended with a call to ethics in one way or another. I wanted to end by asking each of you to articulate the ethical stakes you are most attuned to, with regards to the topic of this dialogue.

TW: Where I try to start and end, is always just asking cis analysts to practice turning their questions of gender back onto themselves, and away from their trans analysands. I also think it’s essential that we become more attuned to the defensive projections that saturate discourses surrounding trans adults and children – within the clinic, psychoanalytic writing, and more broadly to social issues like legal interventions. Childism is one way among many that we can use psychoanalytic theory to consider how prejudice might show up in counter-transference.

LS: I ended my article stating that each child and family is unique. So my wish is that there is more training to work with children in their uniqueness. There’s not a lot of training on even play therapy more generally, and if you don’t play with a child then there is no way of knowing what’s on the child’s mind. So we have a real ethical problem in terms of hearing stories from children, because clinicians are not trained to be with their experience enough. So my ethical call would be to place a value (in training, in the mental health field, and beyond) in listening to the child’s story, with integrity via the way they need to tell it.

OG: I’m reminded it’s not just a set of rules that we follow but it’s a dialogue, and in that way our work is always close to fantasy, with projections and introjections. One of the aspects of ethics involving the analyst is the capacity to contain both the rage that accompanies change – because analysts are now pushed to change their views about gender – and our legitimate fear, because there are fears involved in thinking of gender, our own included.

EW: I focused on how virtue ethics predominates today, particularly in psychotherapy. This ethic seeks to, for example, align body and mind and make promises of happiness. And these promises are destined to fail. This is not the ethic as I see it that orients the psychoanalytic method; the psychoanalytic ethic is oriented to the impossibilities of being, to the very fact that mind and body are impossibly divided but we must find a way somehow to come to terms with this, and in a way that is liveable for each and every one of us. For some of us that is more difficult, and we should be particularly attuned to that. It is interesting to think about what you raise in particular Tobias, which is intransigent attitudes in psychoanalysis broadly toward transgender expressions and gender non-conformity. A psychoanalytic ethic should be oriented toward greater openness, and historically, on this subject, it hasn’t done that and has some way to go.

OG: I just wanted to comment on this idea of alignment, Eve, that you bring up. There is a fantasy that the transsexual believes there is a complete alignment between the body and psyche, but I think that’s a fantasy of people who are not transgender, that this is what the transsexual seeks. I think the transsexual body shows us the capacity to live with contradictions. I haven’t met a transgender or transsexual person who really believes that hormones or surgery is the road to feeling complete. But there is a question of representation in the world, and that is a very, very difficult question – it’s very difficult to live in an unintelligible body. So I think the question of surgery that is posed by many as in opposition to symbolization is an example of splitting in the question itself.

EW: I do hear in my clinical practice this wish to align mind and body, and we hear it also with those who identity as gay and lesbian, and in many others besides. This is very common and while we may be able to interpret it and hear it in its phantasmatic dimension, it has a very concrete aspect out there in the world for people. I think it’s one area where we as analysts can very importantly intervene to open up a space outside of this kind of concretization, a space for thinking about the wish to unify mind and body and what that would satisfy.

## Footnotes

1 Details have been altered to protect confidentiality.

2
https://growinguptransgender.com/2019/08/11/watchful-waiting-a-parents-view/

